# Effective polyethylene glycol passivation for the inhibition of surface interactions of peripheral blood mononuclear cells and platelets

**DOI:** 10.1186/1559-4106-8-14

**Published:** 2013-06-20

**Authors:** Alexander Sauter, Gunther Richter, Alexandre Micoulet, Aurora Martinez, Joachim P Spatz, Silke Appel

**Affiliations:** 1 Department of BiomedicineUniversity of BergenJonas Lies vei 915009BergenNorway; 2 Max Planck Institute for Intelligent SystemsHeisenbergstraße 370569StuttgartGermany; 3 University of HeidelbergINF 25369120HeidelbergGermany; 4 Department of Clinical Science, Broegelmann Research LaboratoryUniversity of BergenLaboratory building 5th floor5021BergenNorway

**Keywords:** Polyethylene glycol, Peripheral blood mononuclear cells, Surface passivation, Unspecific interactions

## Abstract

The inhibition of unspecific adhesion of human white blood cells is a prerequisite for applications requiring the control of defined surface interactions. In this study, a passivation agent based on polyethylene glycol (PEG) for glass surfaces was investigated for the use with human peripheral blood mononuclear cells (PBMC). The grafting of 2000 g/mol methoxy-terminated PEG-urea-triethoxysilane (mPEG2000) onto glass surfaces successfully inhibited unspecific spreading of both human PBMC and platelets in all experiments. The prevention of surface interactions was independent on the anticoagulant used during blood collection. The total efficiency to prevent even transient immobilization of PBMC to the PEG modified surfaces was 97 ± 2%. This makes the passivation with PEG a well suited surface modification for preventing unspecific surface interaction in order to study only defined surface interactions of human PBMC.

**Electronic supplementary material:**

The online version of this article (doi:10.1186/1559-4106-8-14) contains supplementary material, which is available to authorized users.

## Background

A striking difference between in vivo and in vitro systems is the adhesion signalling. There are many important adhesion and homing molecules in immunology [1, 2] and adhesion-molecule signalling is an important factor for the development of specific subpopulations [3]. Control of in vitro systems is a prerequisite for being able to analyse adhesion-mediated signalling. Here, we focused on preventing all unspecific adhesion which might lead to unknown and uncontrollable cell signalling.

As a cellular probe, peripheral blood mononuclear cells (PBMC) were used in this study. PBMC are highly motile cells and play an important role as source of primary immune cells as well as immune cell progenitors. Being a relatively easy accessible cell source, they represent the main cell source for human ex-vivo immunology studies. To date, isolated cells for ex-vivo studies are usually cultured on standardized plastic cell culture dishes, both in immunology and other fields of biology. These dishes are commonly based on polystyrene, with a treated surface to facilitate adhesion and spreading independent of the type of cell. The cell-surface interactions on these culture dishes can be assumed to be relatively unspecific, based on the chemical treatments used to make polystyrene surfaces suitable for cell adhesion, as already investigated by Curtis et al. in 1983 [4]. Integrins, the natural adhesion molecules, play a major role in the adhesion to these synthetic surfaces, mediated by adsorbed proteins like fibronectin, fibrinogen or immunoglobulin G [5], resulting in a rather bio-mimetic but to a major degree uncontrollable surface interaction. One family of molecules, polyethylene glycols (PEGs), has already been investigated for the prevention of interactions with several different cell types for some years now [6]. However, the efficiency to prevent surface interactions of PBMC on PEGylated surfaces remains unclear. PEG-like modified surfaces have been tested with relative success mainly for the repellence of especially serum and plasma proteins, using probes like bovine serum albumin (BSA) [7], fibrinogen [8–10] or foetal calf serum (FCS) [7]. However, activated complement factors might play an exceptional role in mediating adhesion as complement factor 3 has been shown to adsorb even to certain PEGylated surfaces [10, 11]. Especially monocytes, one type of PBMC, are characterized by a distinct adhesion to a variety of different materials and molecules. This is mainly mediated by β_2_ integrins (CD18) expressed by monocytes which have been shown to bind many different ligands [2]. Integrin α_M_β_2_ (CD11b/CD18 or Mac-1) and α_X_β_2_ (CD11c/CD18) bind to surface-bound C3b, both on PEG-like surfaces [11] and other biomaterials [12]. This complement binding might even in serum- and plasma-free conditions aid the adhesion as monocytes are capable of synthesising complement [13]. Additionally, monocytes can produce reactive oxygen species, which can be upregulated upon integrin signalling [14]. These reactive oxygen species might damage the PEG-surface, leading to direct adhesion of the cells, or just alter it enough to permit complement binding, which might indirectly lead to adhesion of the monocytes. In the present study, we therefore aimed to investigate the effectiveness of a PEG-mediated passivation in order to prevent all surface interactions of PBMC in serum-free conditions by use of PEG-monolayers grafted to glass cover slips.

## Methods

### Materials

Glass cover slips were obtained from Carl Roth GmbH & Co. KG (Karlsruhe, Germany). Extran® MA 01, hydrogen peroxide (35%), ethyl acetate, triethylamine and hydrochloric acid (32%) were from Merck KGaA (Darmstadt, Germany) and sulphuric acid (95%) from VWR BDH Prolabo® (Oslo, Norway). Microscope slides (cut edges) were purchased from Gerhard Menzel GmbH (Braunschweig, Germany). mPEG2000-urea-triethoxysilane was synthesized as described by Blümmel et al. [7]. Toluene, obtained from Merck KGaA (Darmstadt, Germany), was dried over a molecular sieve 0.3 nm from Carl Roth GmbH & Co. KG (Karlsruhe, Germany) under nitrogen atmosphere. Methanol, L-glutamine solution (200 mM, BioXtra) and RPMI-1640 Medium (25 mM HEPES modification) were purchased from Sigma-Aldrich (Taufkirchen, Germany) and Lymphoprep™ was purchased from Axis-Shield PoC AS (Oslo, Norway). EDTA tubes (6 ml K2EDTA), heparin tubes (9 ml) and citrate tubes (6 ml CPDA or 9 ml ACD-A) were from Greiner Bio-One GmbH (Kremsmünster, Austria). Sylgard® 184 Silicone Elastomer (poly(dimethylsiloxane); Part A: B 9:1) was obtained from Dow Corning (Midland, USA) and twinsil® 22 (duplicating silicone; Part A: B 1:1) from picodent® (Wipperfürth, Germany). Phosphate Buffered Saline (PBS), without magnesium and calcium, was purchased from Lonza (Verviers, Belgium). ACLA silicon probes were from Applied NanoStructures, Inc. (Santa Clara, USA) and silicon wafers (P-type) from WaferNet, Inc. (San Jose, USA).

### Surface modifications

All steps were performed in the same way for 5 batches of 4 cover slips each. The term ‘batch’ is throughout the study consistently and exclusively used for the modified surfaces, successively named batch 1–5. The glass cover slips were first cleaned in an ultrasonic cleaning unit (Sonorex Digital DK 102 P from BANDELIN electronic GmbH & Co. KG; Berlin, Germany) at 50°C and 100% power for 20 min in a 20% (v/v) solution of Extran® MA 01 and after a thorough wash in double distilled H_2_O (ddH_2_O) once more at 50°C and 100% power for 20 min in ddH_2_O. Subsequently, the cover slips were rinsed again in ddH_2_O and immersed into a freshly prepared 10% solution of hydrochloric acid (HCl) and incubated overnight to resolve interfering surface ions, in particular sodium and potassium ions [15]. After a thorough wash with ddH_2_O, the cover slips were immersed into a freshly prepared piranha solution (30% hydrogen peroxide: 95% sulphuric acid 1:2) at room temperature (RT) for 1 h. This treatment ‘activates’ the surface by covering it with hydroxyl groups [16]. The cover slips were washed extensively with ddH_2_O and blow dried in a nitrogen flow just before the PEGylation step. The one-step PEGylation of the surface was performed as described [7]. In short, dry toluene was added to a reaction flask under a nitrogen atmosphere and the dry and activated cover slips were placed in the toluene. An amount of approximately 0.5 mg of mPEG2000-urea-triethoxysilane per ml of toluene was added, corresponding to 0.25 mM. Triethylamine was then added under nitrogen atmosphere in an amount of 253 nl per ml of toluene (2.5 μM). Approximately 1 μl of ddH_2_O per 10 ml of toluene was added to the inner wall of the reaction flask to aid the reaction (personal communication with Yvonne Schön, MPI Stuttgart, Germany). The reaction flask was closed and incubated at 80°C overnight. The next day, after cooling the flask to RT, the cover slips were directly immersed into ethyl acetate and washed twice in ethyl acetate and three times in methanol. After a sonication in methanol for 2 min at 100%, the cover slips were dried in a stream of nitrogen.

Glass samples for the X-ray photoelectron spectroscopy (XPS) and for the contact angle measurements were prepared as described above. Wafer samples for the XPS were prepared without the HCl step. The samples of intermediate steps were dried in a stream of nitrogen. All samples for XPS and contact angle measurements were stored in a sealed petri dish for about one week prior to measurements.

### X-ray photoelectron spectroscopy

X-ray photoelectron spectroscopy (XPS) was performed in an ultra-high vacuum chamber (DCA Instruments,Turku, Finnland). Al ka (photon energy 1486.6 eV) from a XR 50 twin anode X-ray source (Specs, Berlin, Germany) was used to excite photo electrons. The kinetic energy of the electrons was measured in a hemispherical mirror analyser (Phoibos 150 with 2D CCD detector, Specs, Berlin, Germany). The take-off angle relative to the substrate normal was 30°. Survey spectra were recorded with one point per eV and the single element electron binding peaks were scanned with one point per 0.1 eV. The higher resolution scans were performed for Si 2p (90–116 eV binding energy), S 2s (218–244 eV), C 1s (275–301 eV), N 1s (390–416 eV) and O 1s (525–551 eV). The C 1s, Si 2p and O 1s spectra were analysed quantitatively. Shirley background subtraction was performed using an open access tool (Simple Backgrounds, version 4.1, Sven Tougaard, QUASES-Tougaard Inc.) which uses the algorithm published by Shirley [17]. The peaks were fitted with gnuplot, using first a Gauss function to fit for the rough peak position and then the built-in Voigt function, using the results of the Gauss-fit as starting values for the second fit. The offset in binding energies observed on glass samples due to charging effects was corrected by aligning the SiO_2_ peaks to 103.3 eV (adopted from [7]). The offset was between 3.61-4.58 eV for glass cover slips and 0.08-0.14 eV for the silicon wafer samples. Only adjusted spectra are shown. The FWHM of the fitted Voigt peaks was 2.15 ± 0.36 eV, including all peaks.

Peak areas were transformed into atomic concentrations using the atomic sensitivity factors 0.25 (C 1s), 0.66 (O 1s) and 0.27 (Si 2p) [18]. The attenuation of the silicon and of partially the oxygen peak due to the polymer overlayer was neglected in this part. The error given is the fitting error.

The polymer overlayer thickness was calculated based on the exponential attenuation of the Si 2p peak with the attenuation length (maximal peak height of piranha treated surface relative to maximal peak height of PEG modified surface). The grafting density was estimated by use of the thickness and the bulk PEG2000 density. The data was plotted using gnuplot and aligned and coloured with Adobe Illustrator CS4.

### Contact angle measurements

Static contact angles were measured with an OCAH 200 (Data Physics Instruments GmbH, Filderstadt, Germany). Ten measurements were performed per surface using a drop volume of 0.5-1 μl each. The temperature was 22.9°C at a relative humidity of 40.7%.

### Atomic force microscope imaging

The atomic force microscopy (AFM) was performed with a MFP-3D scanning probe microscope (Asylum Research, Santa Barbara, CA, USA). All images were taken in tapping mode, at RT and in air. One surface per PEGylation-batch was imaged. Control samples included cleaned glass cover slips and hydroxylated (piranha treated) cover slips. At least two randomly chosen positions per surface were imaged, covering areas of 5×5 μm^2^ and 1×1 μm^2^ with a 1024x1024 pixel resolution. All images were recorded at 0.5 Hz speed and were processed with a line flattening of first order and a first order plane fit.

### Assembly of cell chambers for short-term surface tests

Microscope slides were cleaned in an ultrasonic cleaning unit at 50°C and 100% power for 20 min in a 20% (v/v) solution of Extran® MA 01 and after a thorough wash in ddH_2_O again at 50°C and 100% power for 20 min in ddH_2_O. The cleaned microscope slides were blown dry and stored until use. One microscope slide and one cover slip with a modified surface were assembled to one chamber, using the microscope slide as top and the modified cover slip as bottom of the chamber. Four spacers made of cross-linked poly(dimethylsiloxane) of a height of 0.8 mm were used to define the chamber height, using one spacer per cover slip corner. The assembly of cover slip-spacer-microscope slide was sealed with a biocompatible, fast curing silicone-based polymer (twinsil®), leaving two gaps for entry and exit to the chamber.

### Isolation of peripheral blood mononuclear cells (PBMC) using density gradient centrifugation

Peripheral blood samples were collected from 5 different healthy blood donors chosen randomly at the blood bank at the Haukeland University Hospital. The average age was 50 years ranging from 33 to 69 years. Three of five donors were male. The blood groups were A- (two donors), A+, 0- and 0+. For each donor, a set of three blood samples was collected in standardized tubes as follows: one 6 ml EDTA tube, one 9 ml Heparin tube and one either 9 ml ACD-A or 6 ml CPDA tube. The three tubes of each donor were processed in parallel and treated the same way. The three blood samples of donor 1–5 correspond to three of the four surfaces of batch 1–5, respectively. The content of each blood tube was poured in a separate 15 ml centrifuge tube, and filled with PBS up to 12 ml and mixed by turning the tube several times. For the density gradient centrifugation, 6 ml of the blood-PBS mixture was layered carefully on top of 3 ml Lymphoprep™, using a new 15 ml centrifuge tube. After a centrifugation step for 20 min at 160 g and 20°C, the top 2.5 ml were discarded to reduce platelet numbers. The density gradient centrifugation was continued for 20 min at 350 g and 20°C, followed by the collection of the PBMC layer. The cells were washed once with PBS and centrifuged for 8 min (extended to 15 min for batch 4) at 350 g and 4°C, followed by three PBS washing and centrifugation steps, each for 6 min at 200 g and 4°C. Finally, the cell pellet was resuspended in RPMI medium without any additives at 4°C. The volume of RPMI medium was adjusted to be proportional to the initial blood volume leading to a 1.5 times higher concentration of PBMC compared to the donor blood, compensating partly for the losses during the isolation. The cell suspensions were stored for no more than 3 h prior to use at 4–10°C, corresponding to a start of cell observation no later than 7 h after blood donation.

### Experimental time line for cell observations

The image acquisition by light microscope imaging (see below) was started during the first 3 min after adding approximately 250 μl of cell suspension to the assembled cell chamber. Atmospheric temperature and carbon dioxide content were regulated to 37°C and 5%, respectively. The PBMC did sediment during the first 10 min of the time-laps videos. The three time-laps videos per donor were acquired during the following 3–4 h. The order of the samples was chosen randomly.

### Light microscope imaging

The light microscope imaging of PBMC on the modified surfaces was performed at the Molecular Imaging Center (FUGE, Norwegian Research Council), University of Bergen. All videos were taken with a 63× oil immersion objective with a numerical aperture of 1.4 in bright field using a Zeiss LSM 510 Meta microscope system. Images were collected with an AxioCam MR camera connected to the microscope with a 1388×1040 pixel resolution, corresponding to 233.14×174.69 μm^2^. The exposure time was set to 1 ms per frame. The videos were recorded at a speed of 1 frame per second and for easier file handling saved in 12–18 blocks of 5 min pieces containing 300 frames per file. These were then concatenated and reduced for further processing when needed. The position on the surface was centred but otherwise random.

### Time-laps video analysis

The 60–90 min time-laps videos were analysed for cell numbers, single cell spreading behaviour and mobility.

First, the 5 min video pieces of the time-laps video were concatenated to one video. This was watched several times carefully to look for spreading of cells or platelets.

Secondly, both PBMC and platelets were counted in the last frame of the video. The number of PBMC and platelets per μl of blood was calculated based on the dilution, the imaged frame area and the spacer height of the cell chamber.

Finally, the time-laps videos were reduced to one frame per minute and the cells were tracked using ImageJ (version 1.47c, Wayne Rasband, National Institute of Health, USA) together with a public available manual tracking plugin (Fabrice Cordelieres, Institut Curie, Orsay, France). The resulting cell trajectories consisting of the x and y positions in pixels per frame were then processed using a self-written ImageJ macro. This macro converted the pixel values into micrometre values, calculated the radial distance of each cell from its original position over time (Euclidean distance progression) and counted the amount of transiently static cells. A cell was counted as transiently static when it changed its position during a connected period of 30 min for no more than 6 μm (approximated maximal cell radius). Testing for 30 min periods was found to be a functional trade-off between detecting slowly diffusing cells falsely when using too short periods and not detecting shortly static cells for too longer periods. Because of the 30 min criterion, only cells tracked for at least 30 min were included in the analysis, resulting into a range of 14 to 65 analysed cells per video. The trajectories were plotted in ImageJ for visual control during the analysis and then replotted for publication using gnuplot (version 4.6). Adobe Illustrator CS4 was used to align the plots.

The single cell spreading and mobility behaviour was then used to compare the surface passivation between batches.

### Statistical analyses

The statistical analyses were performed using GraphPad Prism (version 5.02), which was also used to plot data. Values are given as median together with the standard deviation if not otherwise stated. Statistical significance was determined using the built-in one-way ANOVA test with a significance criterion of 5%.

## Results

In order to investigate the potential of a PEG-layer to prevent surface interactions of PBMC, PEGylated glass cover slips were prepared. We decided to use mPEG2000-urea since it has been identified to be the optimal PEG coating for SiO_2_-wafers by Blümmel et al. [7]. PEG modified cover slips were characterized using X-ray photoelectron spectroscopy (XPS), contact angle goniometry and atomic force microscopy (AFM). PBMC were isolated from blood donations and time-laps videos of PBMC on the PEGylated surfaces were recorded and analysed.

### Surface characterization

The XPS analysis of the PEGylated cover slips was done in comparison with two sets of control samples. One set of controls consisted of piranha treated and PEGylated silicon wafers and was used to confirm peak positions. The second set of controls consisted of glass cover slips from intermediate steps of the processing, both after the HCl step and after the piranha step, respectively.

**Figure 1:**
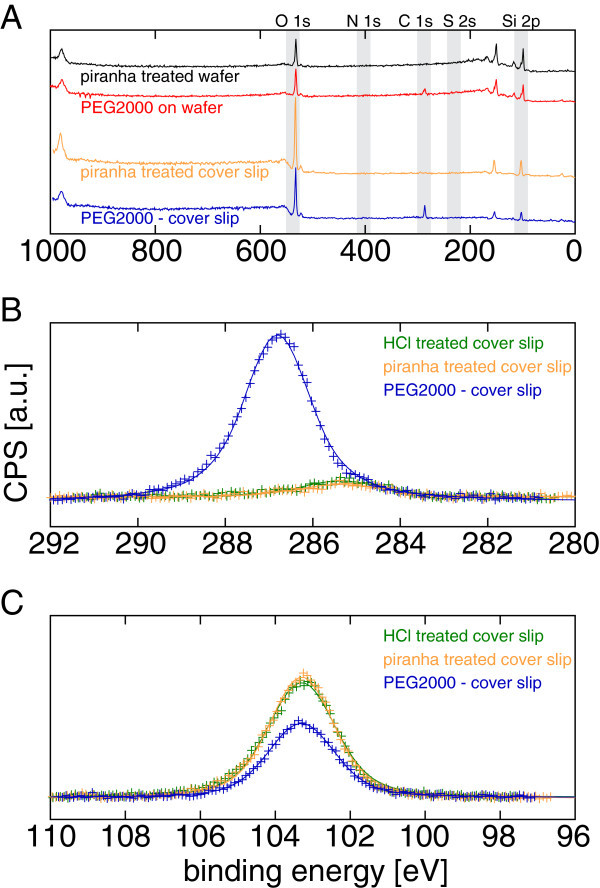
**XPS spectra of modified silicon wafers and cover slips.** (**A**) Survey spectra of piranha treated wafer (black) and PEGylated wafer (red) were used to verify the spectra-alignment of the piranha treated cover slip (yellow) and the PEGylated cover slip (blue). Oxygen and silicon are the only distinct peaks after piranha treatment both for the wafer and the cover slip. After PEGylation, there is an additional carbon peak arising. (**B**) Scanning the C 1s range in higher resolution, nearly no carbon is detected on the cover slips without PEGylation, both after the HCl step (green) and after the piranha treatment (yellow). A distinct peak is measureable after the addition of the PEG layer (blue). (**C**) The silicon dioxide peak at 103.3 eV is the only peak in the Si 2p range. Due to the piranha treatment (yellow), the peak amplitude rises relative to the HCl treated surface (green) slightly. The amplitude decreases considerably due to the attenuation effect from the PEG layer after PEGylation (blue).

XPS survey spectra with the adjusted binding energies are shown in Figure 1A. The corrected peak positions were determined by peak fitting with a Voigt function (asymptotic standard error of the fit was never above 0.07 eV). Piranha treated silicon wafer (offset: -0.08 eV): 285.25 eV for C 1 s, 532.74 eV for O 1 s, 99.54 and 103.30 eV for Si 2p. PEGylated silicon wafer (offset: -0.14 eV): 286.86 and 289.85 eV for C 1s, 532.81 eV for O 1s, 99.62 and 103.30 eV for Si 2p. HCl treated cover slip (offset: 3.86 eV): 285.36 eV for C 1s, 533.17 eV for O 1s and 103.30 eV for Si 2p. Piranha treated cover slip (offset: 4.24 eV): 285.35 eV for C 1s, 533.27 eV for O 1s and 103.30 eV for Si 2p. PEGylated cover slip (offset: 3.46 eV): 286.81 eV for C 1s, 533.03 eV for O 1s and 103.30 eV for Si 2p.

The carbon peaks of HCl treated (green), piranha treated (yellow) and PEGylated cover slips (blue) are shown in Figure 1B. As expectable, there is basically no carbon before the PEGylation but a distinct carbon peak after PEGylation.

The silicon dioxide peaks (Figure 1C) were used to estimate the polymer thickness based on the attenuation of the Si signal due to the polymer overlayer. The attenuation length for mPEG2000 was estimated to be 4.275 nm, using the NIST Standard Reference Database 82 (NIST electron effective-attenuation-length database, version 1.3, distributed by the National Institute of Standards and Technology, USA), and 3.675 nm, respectively, using QUASES-IMFP-TPP2M (Inelastic electron mean free path calculated from the Tanuma, Powell and Penn formula [19], code written by Sven Tougaard, Quases-Tougaard Inc.). We used 3.675 nm since it is closer to the attenuation length used by others (e.g. by Zhu et al. [20]: 3.65 nm), but accounted for the big variation of the estimates in the error calculation. Based on the attenuation length and the calculation outlined in the methods section (formula e.g. explained in [20]), the dry polymer thickness was calculated to be 1.56 ± 0.38 nm. The grafting density can be estimated from the layer thickness when the polymer density is known. We used a density of 1 g/cm^3^ as it has been used by Zhu et al. [20]. Nevertheless, this might be an underestimate as PEG2000 is listed with a density of 1.21 g/cm^3^ at 20°C elsewhere (Merck KGaA, Darmstadt, Germany). We accounted for this in the error calculation. Using the thickness and the density (both conservative estimates), the surface density was determined to be 0.47 ± 0.15 PEG-molecules per nm^2^. As an ideal limit for a monolayer, the cross-section of crystalline PEG in a helical brush-like conformation is given to be 0.213 nm^2^ per molecule [21]. Based on this, the grafting density relative to its theoretical limit can be calculated in analogy to Zhu et al. [20] by dividing the measured area per molecule by the idealized area per molecule. Doing this, we got a grafting density of 10.01 ± 3.24%. Assuming a regular hexagonal distribution, the molecule-to-molecule distance can be calculated from the area occupied per molecule. Taking twice the apothem, the molecule-to-molecule distance was calculated to be 1.57 ± 0.25 nm. Using the model of de Gennes [22] in analogy with Nicholas et al. [23], the polymer thickness in an ideal fluid can be estimated based on the intermolecular distance. With a monomer size of 0.35 nm and a number of monomers of MW/44 = 45.45, the PEG-layer thickness in fluid was calculated to be 5.85 ± 0.95 nm. The Flory radius based on these numbers was calculated to be 3.46 nm. The Flory radius is an estimate of the molecule radius in mushroom conformation, meaning with no overlap between molecules. Thus, based on a circular area per molecule of a radius equal to the Flory radius, the grafting density for a PEG2000 layer with mushroom conformation would be ≤ 0.57%. The grafting density of 10.01 ± 3.24% on our surfaces suggests a sufficiently dense coil-like PEG layer, about 18 times denser than the closest packed mushroom conformation.

**Figure 2:**
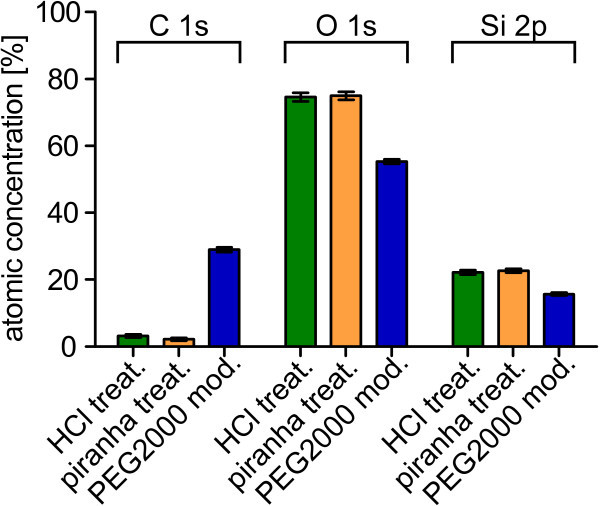
**Atomic concentrations of modified cover slips.** Atomic concentrations do not differ considerably between the HCl treated cover slips (green) and the piranha treated cover slips (yellow). The carbon percentage rises strongly due to the PEGylation (blue). The relative decrease in oxygen and silicon is mainly due to the scaling and the polymer-induced attenuation.

Next, we determined the atomic concentrations from the XPS data. The atomic concentrations were calculated from the peak areas (Figure 2). Not much variation was seen between HCl treated (green) and piranha treated glass cover slips (yellow). A distinct increase in carbon percentage up to a value of 29.0 ± 0.7% upon PEG binding was observed. The perceptual decrease in oxygen and silicon for the PEGylated cover slip is a convoluted effect of the scaling in order to get a total of 100% and the attenuation of the signals as most of the oxygen and silicon is expected to be under the layer of PEG.

**Figure 3:**
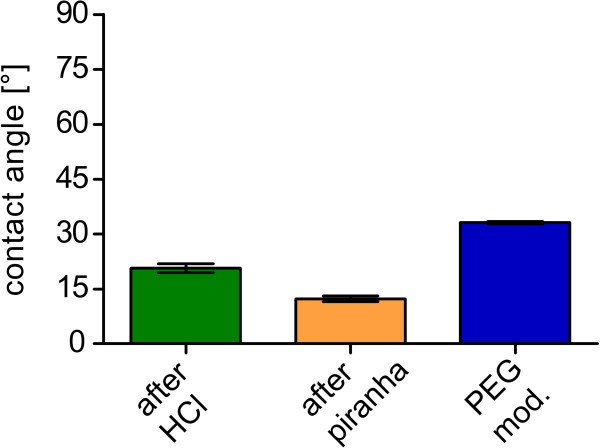
**Contact angles on modified cover slips.** The piranha treatment (yellow) decreases the contact angle relative to the cover slip after HCl treatment (green) from 20.67 ± 1.23° to a value of 12.30 ± 0.81°. The contact angle is increasing due to the PEGylation (blue) to a value of 33.11 ± 0.29°.

We further characterized the surfaces by means of contact angle goniometry (Figure 3). The measured static contact angles were 20.67 ± 1.23° for HCl treated cover slips (green), 12.30 ± 0.81° for piranha treated cover slips (yellow) and 33.11 ± 0.29° for the PEGylated cover slips (blue).

**Figure 4:**
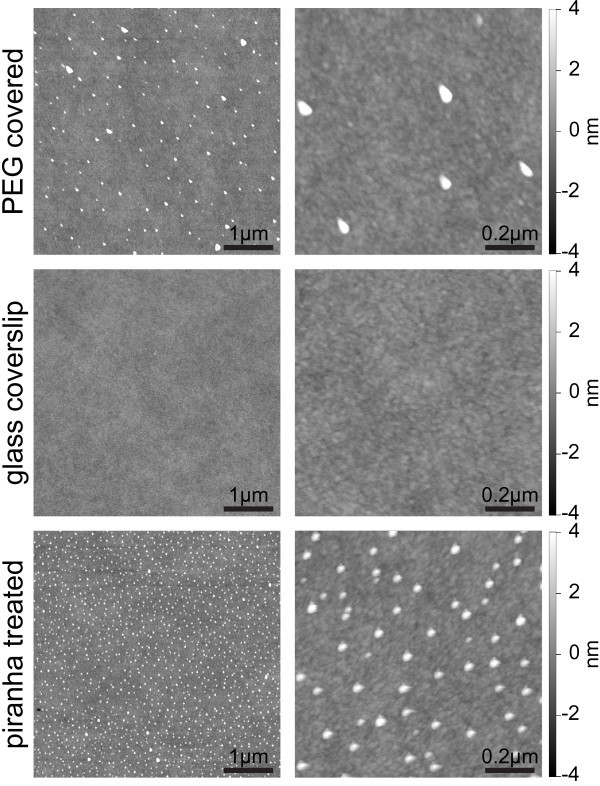
**Representative AFM height profiles acquired in tapping mode.** Images of the dimensions 5 × 5 μm (left) and 1 × 1 μm (right) of a PEG covered glass cover slip (top), cleaned glass cover slip (middle) and piranha treated cover slip (bottom) are illustrated. AFM reveals some defects on PEGylated surfaces.

In order to investigate for possible topological surface abnormalities that could account for punctual passivation defects, AFM pictures were taken from surfaces of the same 5 batches used for cell experiments. Cleaned and piranha treated cover slips served as controls. Representative images are shown in Figure 4. The flatness and homogeneity observed for the cleaned cover slip (middle row) is mainly conserved on the PEG-modified surfaces (top row). However, planarity defects have clearly been introduced, resulting in areas of local height differences relative to the background of > 4 nm. The surface after piranha treatment (bottom row) showed also a defect pattern, but a different one than that after PEGylation (top row). Thus, the defects after the piranha treatment must have either got covered by the PEG-molecules or removed during the PEGylation step. Noteworthy, the XPS data showed no peak in the S 2s range, indicating that there was no residual sulphuric acid detected after piranha treatment.

### Cell isolation yield and purity

**Figure 5:**
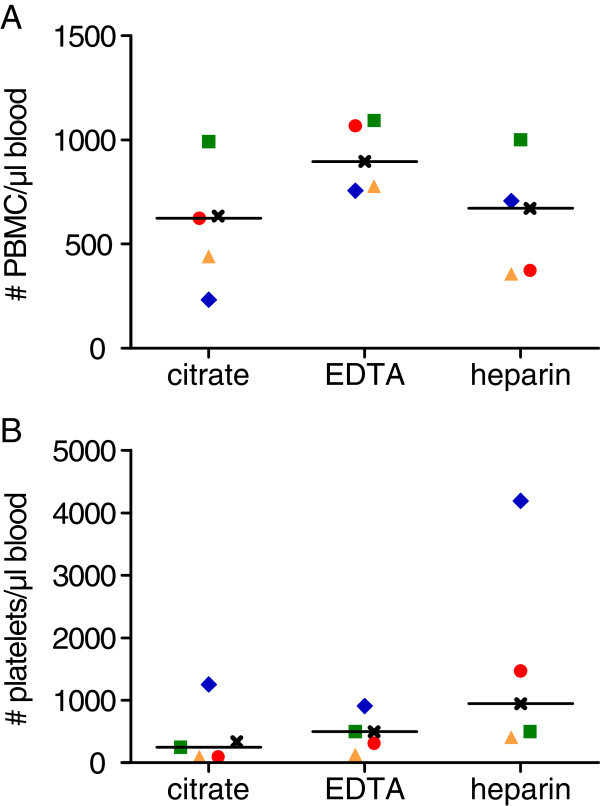
**Cell isolation yield and purity.** (**A**) PBMC numbers and (**B**) residual platelet numbers per μl of blood are shown after isolation with density gradient centrifugation in dependence of the anticoagulant used during blood collection. Each donor is represented by a colour-coded symbol (n = 5). The median is marked with a line. Use of citrate, EDTA and heparin as anticoagulant results in similar numbers of PBMC (**A**) and of residual platelets (**B**).

The median number of PBMC per μl of blood varied only slightly with the anticoagulant used during blood collection and density gradient centrifugation (625 (citrate), 897 (EDTA) and 671 (heparin); Figure 5A). The median number of PBMC including all donors was 707 ± 271 per μl of blood. Notably, the number of PBMC was highest when using EDTA. The median number of residual platelets after the cell isolation process was similar with all three anticoagulants (Figure 5B). As a result of the isolation method, the number of platelets was in the same order of magnitude as the number of PBMC but low enough not to interfere with the observation of PBMC-surface interactions.

### Passivation efficiency for PBMC

**Figure 6:**
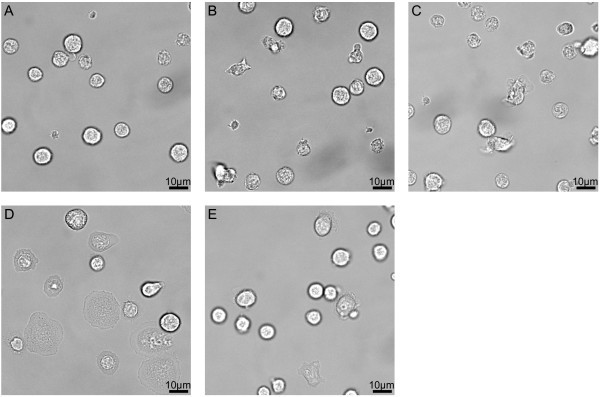
**Representative bright field microscopy images of PBMC on modified surfaces.** The images were taken on PEG-modified surfaces (**A**-**C**), piranha treated cover slips (**D**) and bovine serum albumin (BSA) covered glass (**E**) after 37–54 min (representative of 5 repeats for **A**-**C** and of 3 for **D** and **E**). PEGylation of glass cover slips inhibits spreading of PBMC independent of the anticoagulant used (citrate in **A**, EDTA in **B** and heparin in **C**). Both PBMC and platelets are spreading extensively and irreversibly on piranha treated glass (**D**). On bound BSA (**E**), some cells are spreading with or without migration, while most of the PBMC stay localized without spreading. Platelets attach to neither the PEG surfaces (**A-C**) nor the BSA modified surface (**E**), but do though strongly on glass (not visible on this section).

In order to test for the functionality of the surface passivation for PBMC, we looked closer at the spreading behaviour and the mobility of the single PBMC during the first hour on the surface. A cleaned cover slip was initially used as a control, but all cells, especially the platelets, attached to the glass, showing a rather unnatural looking morphology (Figure 6D and Additional file 1). As a second control, bovine serum albumin (BSA) was used to modify cleaned cover slips. BSA is known to prevent unspecific binding in several other assays and could potentially have been a possible passivation agent also for these cells. Anyhow, cover slips covered with BSA were insufficient at inhibiting adherence of PBMC, reflecting the special adhesion properties of these cells (Figure 6E and Additional file 2). A part of the cells, especially monocytes, were able to spread on these surfaces within very short time after the first surface contact but also most of the other cells showed a rather static behaviour, neither moving by diffusion nor by medium drift. Interestingly, platelets showed no spreading on BSA-modified surfaces and did rarely bind to these surfaces at all.

In contrast to the BSA-modified surfaces, none of the PBMC and none of the platelets showed any spreading on any of the PEGylated surfaces or the different anticoagulants used during blood collection (Figure 6A-C, Additional files 3, 4 and 5). There was no increase in spreading over time for both the cells without and with lamellar protrusion activity.

**Figure 7:**
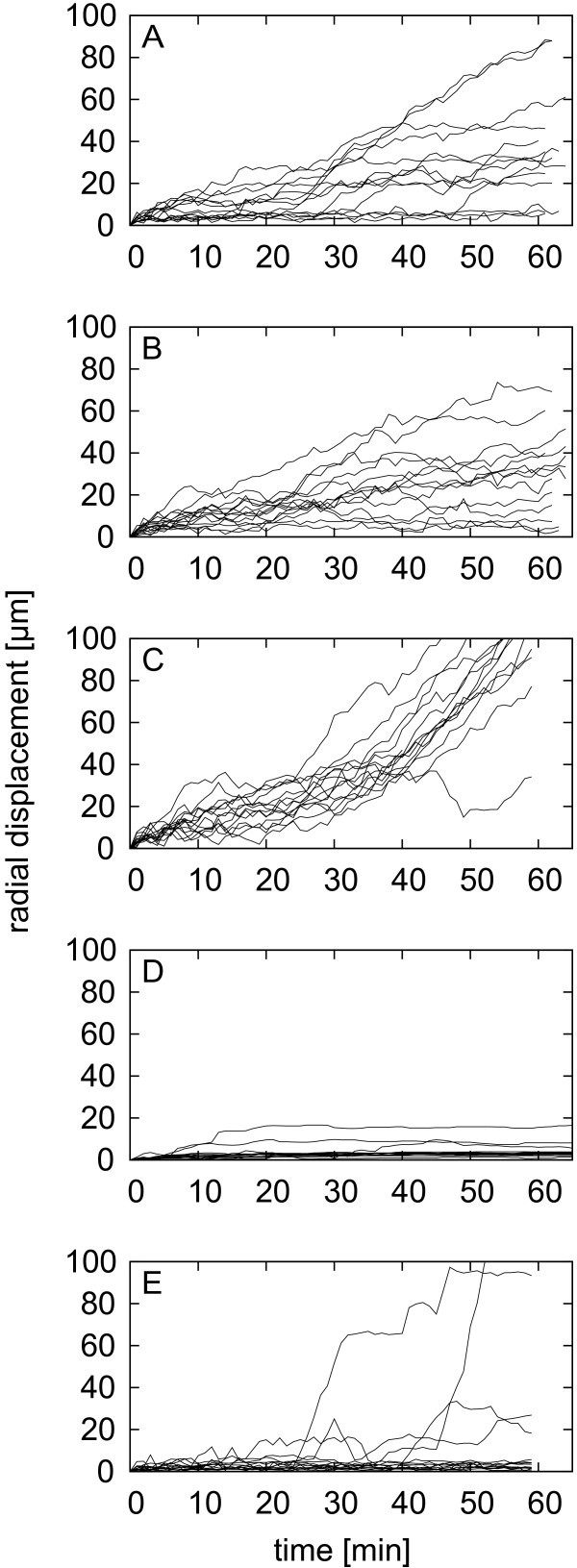
**Radial displacement of PBMC on modified surfaces.** The displacement is plotted over time for three PEGylated surfaces of the same batch (**A**-**C**), a piranha treated cover slip (**D**) and a bovine serum albumin (BSA) covered cover slip (**E**). 14 randomly chosen cells are plotted for each surface. Most PBMC are mobile on PEGylated surfaces (**A-C**), while most are locally fixed on BSA (**E**) and basically all immobilized on glass (**D**).

**Figure 8:**
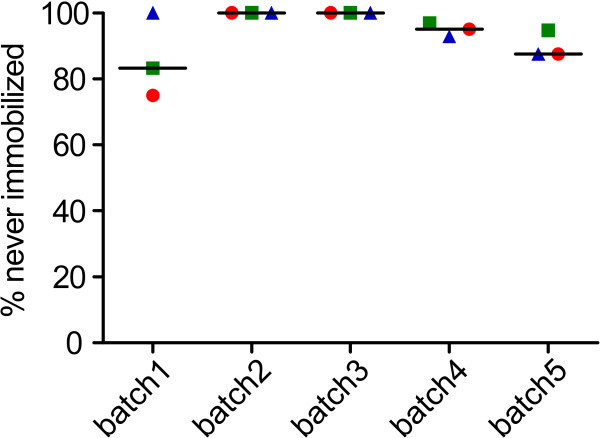
**Efficiency to prevent transient immobilization of PBMC on PEGylated surfaces depending on the production batch.** Red circles: PBMC isolated from citrate treated blood; green squares: PBMC isolated from EDTA treated blood; blue triangles: PBMC isolated from heparin treated blood. The median is marked with a line. PEGylation of glass cover slips efficiently prevents transient immobilization of PBMC with slight variation from batch to batch.

In order to get more detailed information on the passivation efficiency of the single surfaces, we performed a cell-motility analysis of the single cells. The PBMC were tracked digitally in the post-processing of the time-laps videos and the radial displacement from the cells’ original position was plotted over time (Figure 7). The radial displacement is, similar to the mean squared displacement in Brownian motion, expected to increase in average or at least vary over time for all cells without stable surface interaction, in part due to diffusion and in part to a residual medium drift, comparable to an analysis performed by Qian et al. [24]. For most of the cells on PEGylated surfaces, this expectation was confirmed (Figure 7). However, some cells on some surfaces were static for parts of the time or up to the whole of the observation time, giving rise to horizontal lines in the radial displacement graphs (Figure 7). Notably, even these cells did not spread on the PEGylated surfaces. In order to quantify this transiently static behaviour of PBMC on PEG surfaces, the percentage of cells being immobilized over more than 30 min was determined. The percentage of never immobilized cells was plotted depending on the surface batch prepared, resulting in a passivation efficiency for each batch (Figure 8). No statistically significant difference could be observed between the passivation efficiencies of the different PEGylation batches. The total efficiency to prevent the transient binding of PBMC to PEG surfaces was 97 ± 2% (median ± SE), while the prevention of spreading was 100% for all experiments on PEGylated surfaces.

## Discussion

In the present study, we show that PEGylation of glass surfaces successfully inhibits unspecific spreading and to a major extent even transient surface-interactions of human PBMC and platelets, despite topological defects seen in AFM. This effect was independent of the anticoagulant used during blood collection citrate, EDTA and heparin, respectively. Cell numbers differed only slightly with the use of different anticoagulants during blood collection.

### Surface characterization

In order to characterize the quality of the PEG layer, we used XPS, contact angle goniometry and AFM for the investigation of the modified surfaces. While XPS and contact angle data are expected to give an average picture of the surface composition, AFM was used to look at the local homogeneity.

The XPS carbon peaks of PEGylated surfaces were measured at binding energies close to 286.8 eV, which can be correlated to oxygen-bound carbon [7], confirming the binding of ether-rich PEG. This is similar to what others have observed [20]. The largely increased area under the curve is correlated with the increased amount of carbon due to PEG binding, further confirming the success of the surface modification. The small carbon peaks on surfaces without PEG close to 285 eV (Figure 1B) might indicate a small, probably airborne, hydrocarbon contamination, as this is the typical binding energy of aliphatic carbon [25].

In order to be able to compare our data with other studies, we determined both the layer thickness and the grafting density as a measure of quality. The PEG-layer thickness of 1.56 ± 0.38 nm is close to the value of 2.15 ± 0.20 nm published by Blümmel et al. using the same method [7], suggesting a similar surface quality. The surface density of 0.47 ± 0.15 PEG-molecules per nm^2^ on our surfaces is similar to the value of 0.4 molecules per nm^2^ for the highest density region of a PEG2000 density-gradient based on silane-binding published by Lin et al. [26]. In the same study they further measured the contact angle in the highest density region to a value of 34° for the advancing contact angle and 24° for the receding. The contact angle of our surfaces of 33.11 ± 0.29° is in the range between those, suggesting a comparably high PEG density. Notably, the contact angles might have been lower for HCl and piranha treated surfaces directly after preparation. However, even after several days, allowing a certain recovery, the contact angles still differed strongly (Figure 3).

The grafting density in our study was calculated to be 10.01 ± 3.24%. In general, long polymer chains are far less probable to extend fully than small ones. Thus, in analogy to what has been shown by Zhu et al. [20], PEG-molecules of a molecular weight much higher than 300 and bound in one step will always have a grafting density far less than 100%. This was demonstrated with a method were the polymer binding is less sterically hindered than with the triethoxysilane used in our study. Even under those conditions, they measured a grafting density not higher than 35% for PEG2000 and concluded to have a coil-like PEG conformation [20]. The about two third lower grafting density in our study, despite the sterically bigger linker, accounts for a high quality and obviously gave a satisfactory result in passivation. In general, the passivation efficiency for long PEG-chains might only vary insignificantly in a broader range of grafting density since the coil-like PEG conformation can compensate for the density variations as long as the density does not decrease to a mushroom-like conformational regime, where a sufficient coverage of the substrate cannot be expected anymore.

### Cell isolation yield and purity

In our study, we used a modified density gradient centrifugation method for the isolation of PBMC. The number of cells was adjusted for the experiments, so that there was no impact of the isolation yield on the experiment. However, the number of PBMC including all donors being 707 per μl of blood (95% reference range: 558-859/μl) was considerably lower than reference numbers for Caucasians of 1140-3820/μl, originating by adding monocyte numbers (140-620/μl) to lymphocyte numbers (1000-3200/μl) [27]. The isolation yield can be calculated to a value of 33%, based on the mean number of monocytes plus lymphocytes from [27] and the median number of PBMC after isolation in our experiments. The considerable loss in PBMC using density gradient centrifugation might be a major limitation of the method in cases when the cell numbers are crucial. The reduction of platelets, on the other hand, was, with a remaining number of only 0.2% relative to the reference value for Caucasians [27], quite efficient.

### PBMC preparation

As a major influence on the cells during isolation, the effect of the anticoagulant used was analysed in this study. The Ca^2+^ chelating agents, citrate and EDTA, might inhibit certain pathways necessary for certain cell-surface interactions. Heparin might lead to activation or strengthening of adhesion due to its integrin-binding property and might therefore influence the passivation efficiency negatively. However, no significant difference in passivation efficiency was seen depending on the anticoagulant used during cell isolation.

Interestingly, we observed that monocytes isolated from heparin blood showed more often lamellar searching protrusions compared to cells isolated from citrate or EDTA blood. One possible explanation for this phenomenon might be heparin binding to the integrins α_M_β_2_ (CD11b/CD18) and α_X_β_2_ (CD11c/CD18), since heparin is a known ligand of those [2] and might stimulate the cells through the connected pathways. However, a quantification of this effect was not performed in this study.

### Passivation efficiency for PBMC

The main aim of this study was to test the effectiveness of a PEG-based glass modification in preventing PBMC adhesion and spreading, and in that respect our modified surfaces were found to be satisfactory. We observed a 100%-efficiency in preventing all cell spreading, as there was no cell spreading on any of the PEGylated surfaces despite the distinct adhesion properties of PBMC. Our results are in agreement with those of Blümmel et al. who found a reduction in cell numbers for fibroblasts on surfaces likewise modified with mPEG2000-urea of 0.006 ± 0.030 relative to a Petri dish control [7]. One possible explanation for the slightly lower efficiency in preventing stable cell spreading observed by Blümmel et al. might be the secretion of extracellular matrix onto the surface by the fibroblasts. In the mentioned study, the fibroblasts were cultured on the surfaces for 48 h, so an increasing amount of extracellular matrix proteins might have formed some adhesion spots on top of PEG-surface-defects, similar like the defects seen in our AFM images.

In contrast to most other studies, we did not reduce the test for surface interactions to single time point-observations of spreading behaviour but included in the quality analysis of the surfaces also the transient immobilization without spreading. The transient immobilization could be shown to be very limited on these PEG surfaces, representing a nearly perfect passivation for PBMC. Interestingly, the differences between batches were bigger than intra-batch differences (Figure 8). Based on this, the variation of the passivation efficiency seems to depend mainly on the preparation of the surfaces, possibly including variations of parameters not controlled in this study. The topological defects observed in the AFM images might be an indication of the remaining potential of optimization of the surfaces. Being otherwise relatively homogeneous, these defects might be the reason for the transient immobilization of PBMC on these surfaces. It has to be kept in mind that the AFM images were taken in air. In fluid, the PEG-molecules should rise and possibly cover at least a part of the defects seen in the AFM images, especially since the diameter of the defects is in fact less than that indicated by the AFM analysis. This is due to the nature of AFM imaging, where the width of the object is increased by the diameter of the tip, as e.g. discussed by Biro et al. [28]. Nevertheless, it might still be worth finding the source of the inhomogeneity in order to improve the surface quality. A certain clotting of the PEG-silane molecules might be a possible source of the defects, either during storage of the compound or during the PEGylation. To date, it remains unclear if this can be prevented or if it is an unavoidable part of the surface preparation.

## Conclusions

The PEG mediated passivation was efficient for PBMC and independent of the choice of anticoagulant used during blood collection. Even transient immobilization of PBMC could be prevented in 97 ± 2% (median ± SE) of the cases. The anticoagulant heparin influenced the lamellar activity of monocytes. As EDTA irreversibly chelates Ca^2+^ ions, cells isolated from EDTA blood are considered not to be suitable for functional assays. Therefore, we suggest citrate based anticoagulants as preferred choice for the isolation of PBMC in similar assays.

Additional file 1: **Representative time-laps video section of PBMC isolated from EDTA treated blood on a glass cover slip cleaned with piranha solution (representative of 3 repeats).** Both PBMC and platelets are spreading extensively and irreversibly, partly leading to cell death. (MP4 8 MB)

Additional file 2: **Representative time-laps video section of PBMC isolated from EDTA treated blood on a glass cover slip with covalently bound BSA (representative of 3 repeats).** BSA treatment inhibits platelet spreading but not PBMC spreading. (MP4 7 MB)

Additional file 3: **Representative time-laps video section of PBMC isolated from citrate treated blood on a PEGylated glass cover slip (representative of 5 repeats).** PEGylation of the surface inhibits spreading of PBMC isolated from citrate blood. (MP4 7 MB)

Additional file 4: **Representative time-laps video section of PBMC isolated from EDTA treated blood on a PEGylated glass cover slip (representative of 5 repeats).** PEGylation of the surface inhibits spreading of PBMC isolated from EDTA blood. (MP4 7 MB)

Additional file 5: **Representative time-laps video section of PBMC isolated from heparin treated blood on a PEGylated glass cover slip (representative of 5 repeats).** PEGylation of the surface inhibits spreading of PBMC isolated from heparin blood. (MP4 8 MB)
